# The Role of the Lymphocyte Functional Crosstalk and Regulation in the Context of Checkpoint Inhibitor Treatment—Review

**DOI:** 10.3389/fimmu.2019.02043

**Published:** 2019-09-06

**Authors:** Barbara Seliger

**Affiliations:** Institute of Medical Immunology, Martin Luther University Halle-Wittenberg, Halle (Saale), Germany

**Keywords:** T cells, tumor growth, tumor microenvironment, microbiome, inflammation, checkpoint inhibitors

## Abstract

During the last decade, the dynamics of the cellular crosstalk have highlighted the significance of the host vs. tumor interaction. This resulted in the development of novel immunotherapeutic strategies in order to modulate/inhibit the mechanisms leading to escape of tumor cells from immune surveillance. Different monoclonal antibodies directed against immune checkpoints, e.g., the T lymphocyte antigen 4 and the programmed cell death protein 1/ programmed cell death ligand 1 have been successfully implemented for the treatment of cancer. Despite their broad activity in many solid and hematologic tumor types, only 20–40% of patients demonstrated a durable treatment response. This might be due to an impaired T cell tumor interaction mediated by immune escape mechanisms of tumor and immune cells as well as alterations in the composition of the tumor microenvironment, peripheral blood, and microbiome. These different factors dynamically regulate different steps of the cancer immune process thereby negatively interfering with the T cell –mediated anti-tumoral immune responses. Therefore, this review will summarize the current knowledge of the different players involved in inhibiting tumor immunogenicity and mounting resistance to checkpoint inhibitors with focus on the role of tumor T cell interaction. A better insight of this process might lead to the development of strategies to revert these inhibitory processes and represent the rational for the design of novel immunotherapies and combinations in order to improve their efficacy.

## Introduction

It has been generally accepted that the development and progression of tumors is a result of an altered crosstalk between the tumor and the host immune system (1–3). The immune system not only suppresses tumor growth by destroying tumor cells or inhibiting their outgrowth, but also promotes tumor progression by either selecting for tumor escape variants or by establishing conditions within the tumor microenvironment (TME) and periphery that facilitate tumor outgrowth, which has been classified as a hallmark of cancer (4). These include an increased frequency of immune suppressive cells, metabolites, cytokines and soluble factors, hypoxia and acidic pH (5, 6). Further changes of the TME during neoplastic transformation are a selective ablation of immune effector cells and deletion or neutralization of cytokines, like interferon (IFN)-γ (7). Despite interferon (IFN)-γ exert pro-tumorigenic effects under certain circumstances dependent on the cellular and molecular context (8, 9), it represents a key mediator of immunosurveillance produced by natural killer (NK) cells and T cells known to promote cytotoxic activity of macrophages and enhance the expression of immune modulatory molecules on tumor cells (7). This results in the release of tumor associated antigens (TAA) for cross presentation by dendritic cells (DCs), which uptake and process these antigens into peptides then presented via the major histocompatibility complex (MHC) class I and class II molecules to CD8^+^ and CD4^+^ T cells, respectively. However, elimination of transformed cells can be incomplete due to a decreased tumor immunogenicity (10). This results first in an equilibrium state characterized by a balance between proliferation and killing of tumor cells by CD8^+^ T cells thereby maintaining the tumor at a subclinical stage, followed by the generation of tumor cells, which are resistant to immune rejection due to constant selective pressure of the immune system (2). These immune escape mechanisms are associated with the loss or downregulation of TAA and/or HLA class I surface molecules or aberrantly expression of the non-classical HLA-G and HLA-E antigens as well as co-inhibitory molecules (Table 1). This might be at least partially mediated by the induction of oncogenic pathways (11, 12) and changes in the tumor cell metabolism (13, 14).

**Table 1:** Immune escape mechanisms.

**Tumor**	**Microenvironment**	**Periphery**
HLA I ↓	Suppressive cytokines ↑	CTL frequency and function ↓
HLA-G/-E ↑	Suppressive metabolites ↑	NK cells, frequency, and function ↓
IFN signaling ↓	pH ↓	DCs, frequency and function ↓
Oncogenic signaling ↑	Hypoxia ↑	TAN, TAM ↑
iCP ↑	Microbiome	Treg, frequency ↑
Metabolism ↑		MDSC, frequency ↑

↑ *upregulation*, ↓ *downregulation*.

However, interventions, such as chemotherapy, radiotherapy (RT), physico-chemical, and thermal ablation can promote the release of TAA and might overcome the dominant immune suppressive pathways leading to an increased immunogenicity (15–18). Therefore, the combination of immunotherapies with other strategies offers novel opportunities to recover immune activity and increase their efficacy, which result in a better patients' outcome. Indeed, this approach is currently investigated in a number of experimental models and clinical trials (19).

Players involved in mounting anti-tumor immune responses include in particular cells of the adaptive immune system, which protect and/or control tumor outgrowth and the interaction of the host against viral/pathogen infections and neoplastic transformation. The therapeutic potential of host-vs.-tumor activity has been analyzed by various groups and is based on CD4^+^ and CD8^+^ T cell responses, which are part of the cancer immune cycle and significantly influence the clinical outcome of patients (20, 21). It is well-known that the initial antigen-mediated activation of T cells is modulated by the engagement co-stimulatory signals with its ligands on antigen-presenting cells (APC). Under physiologic conditions, immune checkpoint pathways avoid auto-immunity by inducing inhibitory pathways important for maintaining self-tolerance thereby regulating the type and magnitude of T cell responses required to mount a proper anti-tumoral activity. During the last decade, a number of different inhibitory T cell and non-T cell iCP pathways have been well-characterized (Table 2) (31). The prototype is the cytotoxic T lymphocyte antigen 4 (CTLA-4; CD152), which competes with CD28 for the ligands CD80 and CD86, and antagonizes the T cell receptor (TCR) signaling (32–34). In addition, the interaction of the programmed cell death protein 1 (PD-1; CD279) with its ligands the programmed cell death 1 ligand 1 (PD-L1; CD274/B7-H1) and/or PD-L2 (CD273/B7-DC), negatively interferes with TCR signaling (35–38). Thus, immune checkpoints (iCPs) have either a stimulatory or inhibitory potential, the latter acting as “breaks” on the immune response. Recently, there exists evidence that inhibitory iCPs could be targeted by immune check point inhibitors (iCPIs) leading to an increased anti-tumoral response and patients' survival (39).

**Table 2:** T cell associated inhibitory immune checkpoint pathways.

**Molecule**	**Expression**	**Ligand/ receptor**	**Effect**
LAG-3 (CD223)	T and NK cells, TILs	MHC II	Negative regulator of T cell function and DC (22, 23)
TIM-3	NK cells, macrophages	Multiple: SCTLACAM-1, galectin	Induction of MDSC, negative regulator of T cell function (24)
VISTA (PD-L1H)	Tumor, myeloid cells, T cells, Tregs		T cell suppression (25)
TIGIT	T and NK cells	CD155, CD112	Immune suppression IFN-γ↓, IL-10↑ (26, 27)
B7-H3 (CD276)	Tumors, APC, NK, B and T cells	Receptor not yet analyzed	Activation/suppression of T cell and NK cell function (28)
BTLA-4 (CD272)	Tumor cells	HVEM	Inhibition of T and B cell activation
A2aR and CD73	T cells, APC, NK cells, endothelial cells	Adenosine	Attenuation of inflammation, inhibition of T cell activation (29)
B7-H4 (B7x)	ACP, DC, macrophages, tumor cells	Receptor not yet identified	Inhibition of T cell function and differentiation (30)

↑ *upregulation*, ↓ *downregulation*.

## General Strategies of Tumors to Facilitate Tumor Suppression by Analyzing the Composition and Frequency of Immune Cells in the Tumor Microenvironment (TME) and Peripheral Blood (PB)

The impaired anti-tumoral immune response represents an important hallmark of solid tumors and hematopoietic malignancies and involves many distinct mechanisms at the tumor site, in the tumor microenvironment (TME) and in the peripheral blood (21). The generation of an inflammatory and immune suppressive milieu in the TME induces tumor escape mechanisms, such as downregulation of classical HLA class I antigens and an upregulation of HLA-G and -E as well as iCPs including e.g., PD-L1 in the TME and CTLA-4 in the lymphoid tissues leading to evasion of adaptive immune responses (40–42). Furthermore, an upregulation of other immune inhibitory molecules like PD-1, T cell immunoglobulin and mucin domain-3 (TIM-3), lymphocyte-activation gene 3 (LAG-3), 2B4, and T cell immunoglobulin and immunoreceptor tyrosine-based inhibitory motif (TIGIT) have been reported, which is accompanied by a reduced IFN-γ and TFN-α secretion of T cells (43–45). An altered TME is further characterized by an altered cellular composition and activity of tumor infiltrating immune cells. Next to a reduced frequency and activity of immune effector cells, such as CD8^+^ T cells, NK cells, an increased frequency of immune suppressive cells, such as tumor associated neutrophils (TANs), myeloid-derived suppressor cells (MDSC), tumor associated macrophages (TAM), tumor associated fibroblasts (TAF), regulatory T cells (Treg), and stroma cells leading to a complex interaction network of heterogeneous immune and non-immune cell populations with overlapping and opposite functions (46). In addition, soluble factors, like the transcriptional growth factor (TGF)-β, interleukin (IL)-10, the vascular endothelial growth factor (VEGF)A, chemokines as well as metabolites, e.g., arginase, hypoxia, and low pH, have been identified to be responsible for the establishment of an immune suppressive TME (47, 48). Furthermore, a reduced frequency and impaired function of effector cells and capacity of dendritic cells to present antigen as well as an increased number of immune suppressive cells were also found in peripheral blood. Both an immune suppressive TME and a reduced immune function of PB are associated with a poor patients' outcome (49–52). There exists evidence that functional T cell responses could be missed by analyzing only PBL (53) and no TIL. Therefore, it is essential to determine the composition, organization and function of the TME and PB of individual patients as well as the tumor itself to predict potential anti-tumoral effects of antigen specific T cells, since this has been shown to have prognostic relevance and therapeutic implications (47, 54).

## Immune Checkpoint Inhibitors and Patients' Response

Novel immunotherapeutic approaches have recently revolutionized the treatment of solid and hematopoietic tumors. The clinical success of monoclonal antibodies (mAb) directed against CTLA-4 and the PD-1/PD-L1 pathway was a breakthrough achievement (55–57). The anti-CTLA-4 mAb Ipilimumab was the first iCPI approved by the Food and Drug Administration (FDA) (58, 59) followed by the approval of the anti-PD-1 mAbs Pembrolizumab and Nivolumab in 2014 or 2016, respectively. The anti-PD-L1 mAbs Durvalumab, Atezolizumab, and Avelumab were FDA approved in 2017 after promising results in non-small cell lung cancer (NSCLC), urothelial carcinoma and Merkel cell carcinoma (60–63).

Despite the rapid progress of approvals for iCPI, the accumulated experience demonstrated that approximately only one-third of patients had a durable response upon single iCPI treatment. Thus, the majority of patients do not benefit from iCPI alone, which might be due to primary, adaptive and acquired resistance mechanisms (64). Therefore, a number of clinical trials using iCPIs across all tumor types using different combinations, e.g., chemotherapy, iCPIs, chimeric antigen receptor, hypermethylating agents, CDK4 inhibitors, RT and targeted therapies, are currently conducted. Some of these combinations have achieved response rates over 50% (57, 65, 66). Regarding the combination of RT with iCPI it is noteworthy that RT could not only stimulate immune responses, but could also exert immune suppressive effects (67, 68). In this context, scheduling of iCPI therapy is important for the therapeutic outcome in combination with RT (69, 70), which has been shown to shape the T cell receptor repertoire of TIL (71). However, there is still an urgent need to explore biomarkers to predict response to these treatments and to identify combinations of agents to improve treatment efficacy, overall survival (OS) of patients and mitigate toxicities of these treatment options.

## Immune Modulatory Molecules and their Relevance for T Cell Responses and Patients' Outcome

### Expression of Classical and Non-classical HLA Class I Antigens

This topic has been reviewed and discussed by various authors (72–75). HLA class I surface expression is frequently downregulated or lost in solid and hematopoietic tumors. These abnormalities have functional relevance, since they impair T cell recognition of tumors. Furthermore, HLA class I alterations have been associated with a worse patients' outcome and a reduced overall survival (OS) and play a role in the resistance to iCPI therapy. The underlying molecular mechanisms of impaired HLA class I surface expression are diverse and often associated with deficiencies in the expression of components of the antigen processing machinery (APM) and IFN pathways as recently summarized (72, 76). This could be due to either structural alterations or deregulation at the transcriptional, epigenetic or posttranscriptional level of these molecules (77). Furthermore, HLA class I expression has been associated with an increased density of tumor infiltrating lymphocytes (TIL) and an increased anti-tumoral T cell response (78).

Next to the impaired expression of HLA class I antigens, a frequent overexpression of HLA-G and/or –E was found in tumors of distinct origin, but not in adjacent normal tissues or in healthy controls. This was accompanied by a reduced T cell and NK cell recognition and a bad patients' prognosis (79–83). Soluble HLA-G levels (sHLA-G) were also frequently detected and inversely correlate to numbers of activated T cells suggesting that sHLA-G promotes tumor immune escape through activation of immune responses (84).

### Expression of Immune Checkpoints: Challenges and Pitfalls

Next to alterations of HLA class I antigens, high expression levels of co-inhibitory checkpoints, such as e.g., PD-L1 and B7-H4, in various tumor entities are often associated with the clinical outcome of cancer patients (85–88). An altered expression pattern of PD-L1 was found in primary and metastatic bladder tumors suggesting a dynamic nature of the TME (89). The capacity of the PD-L1 mediated immune suppression was inversely proportional to the antigenicity of the tumor (90). Since PD-L1 expression on both tumor and host's immune cells could lead to escape from immune surveillance, PD-L1 expression has been suggested as a biomarker for prediction of prognosis and response to iCPI (91). Its expression correlates with the adaptive immune resistance in several tumor types, including melanoma, NSCLC, Merkel cell carcinoma, breast cancer, mismatch-repair deficient tumors, and Hodgkin's lymphoma (91–93). However, PD-L1 expression does not reliably predict response to iCPI. In melanoma, tumor PD-L1 expression showed a significant correlation with response to five out of eight iCPI studies treating patients with anti-PD-1 mAb, while it did not predict response to anti-CTLA-4 therapy (94). Furthermore, some patients negative for PD-L1 expression can have a response to iCPI (92). In NSCLC, no association of PD-L1 expression with response has been reported with Nivolumab, while PD-L1 expression on at least 50% of NSCLC lesions almost doubled the response rate to Pembrolizumab from 19 to about 45% (95). In contrast to pre-treatment biopsies, tumor biopsies in early treatment phase obtained from metastatic melanoma patients treated sequentially receiving CTLA-4 and PD-1 iCPI showed high PD-1 and PD-L1 expression levels in responders (96). In NSCLC cells, the PD-L1 genomic locus amplification correlated with PD-L1 expression and anti-tumor responses (97, 98). Despite a significant heterogeneity was observed, higher levels of CTLA-4 and PD-L2 expression were found in melanoma patients, who benefit from CTLA-4 antibodies (99, 100). In contrast, PD-L1, PD-L2, and CTLA-4 expression did not correlate to anti-PD-1-responsiveness of melanoma patients (101). In addition to the discrepant results on the role of PD-L1 expression for prognosis and iCPI response, there exist some limitations regarding the analysis of the PD-L1 expression, including membranous vs. cytoplasmic expression, expression by multiple cell types in the TME, focal expression in tumor samples, changes in the expression during disease progression, upon radiation, chemotherapy, and epigenetic drugs and in particular the variability of laboratory techniques and anti-PD-L1 antibodies employed for immunohistochemistry (IHC) (102).

### Somatic Mutations and Neoantigen Load

Increasing evidence demonstrated that mutations lead to the generation of neoantigens, which are presented by HLA class I molecules and can be recognized by CD8^+^ cytotoxic T cells (CTL) (77). Thus, the tumor mutational burden (TMB) might be correlated with the level of response to T cell based immunotherapies. Indeed, a systemic review of melanoma patients showed that responses to iCPIs correlated with TMB, neoantigen load, and immune-related gene expression (103, 104). Microsatellite instable (MSI) colorectal carcinoma (CRC) has large mutational burdens, higher immune cell infiltration and higher response rates to PD-1 blockade (105). However, a high TMB does not always predict responders to iCPI therapy, which might be due to neoantigen heterogeneity and an extremely diverse array of somatic mutations (106, 107). It is noteworthy that T cell epitopes have a similarity to bacterial and viral antigens suggesting a cross reactivity of T cells to intestinal bacterial and viral antigens, which can also modulate the iCPI therapy (108). In addition, PD-L1 expression can be controlled by driver mutations and oncogenic signaling (109–112).

### Immune Profiling Signatures and iCPI

Genetic and immune heterogeneity was found in melanoma responding to immunotherapy. Mutanome and individual gene-based expression analysis demonstrated mesenchymal and T cell suppressive inflammatory or angiogenic tumor phenotypes, which were associated with innate anti-PD-1 resistance (113). Genes, which were higher expressed in non-responding pre-treatment tumors, include molecules involved in epithelial to mesenchymal transition (EMT), immune suppression and chemotaxis of monocytes and macrophages (114). Interestingly, a dormant TIL phenotype characterized by an elevated TMB and intra-tumoral CD3 signal, elevated TILs with low activation and proliferation was associated with a favorable response to iCPI (115). The IFN signature is correlated with an improved prognosis and iCPI response or resistance to iCPIs (116). Furthermore, T cell diversification reflects antigen selection in the blood of patients on iCPI treatment (117). Recently, a 15-gene pre-treatment classifier model was identified to predict response to anti-CTLA-4 treatment (118).

## Role of Immune Cell Subpopulations for Tumor Immunity and the Effect of iCPI

### Tumor-Infiltrating Cytotoxic Lymphocytes (CTL) and iCPI

The success of checkpoint blockade depends on the presence of TIL, particularly of CD8^+^ CTL, in the TME. These CTL are located at the invasive tumor margin and intratumorally, and are negatively regulated by the PD-1/PD-L1-mediated adaptive immune resistance (119, 120). In metastatic melanoma, the presence of CTL at the tumor margin predicted better response to iCPI. Colon cancers with MSI are highly infiltrated with T cells, particularly with CTL, relative to microsatellite-stable (MSS) colon cancers (121–123). Members of the CCL and CXCL chemokine families have been associated with T cell recruitment to melanoma metastases (124, 125). Higher levels of CCL2, CXCL4, and CXCL12 have been noted in tumors responding to iCPI therapy (126).

So far, it is not clear whether CD8^+^ effector memory cells might explain the durable response observed in many patients. Interestingly, brisk CTL infiltrates at time of progression in patients on iCPI treatment were observed suggesting an impaired activity of effector immune cells in the TME leading to therapeutic resistance. Despite CD8^+^ T cell responses against tumor cells are well-understood, information about the role of CD4^+^ T cell immunity in cancer is limited. Tumor specific CD4^+^ T cells have a broad activity beyond the provision of helper signals to CD8^+^ T cells (127, 128). CD4^+^ T cells exhibit anti-tumor effects and Th1 T cells are involved in the killing of tumor cells by secretion of cytokines that activate death receptors on tumor cells and induce epitopes spreading. Furthermore, CD4^+^ Th1 T cells can activate DC functions. The secretion of IL-4 from CD4^+^ T cells could establish long-term memory immune responses and further recruit eosinophils and macrophages.

### Tumor-Infiltrating Regulatory T Cells (Tregs)

Tumor-infiltrating CD4^+^ Tregs were frequently detected in the TME and suppress CTL activity leading to reduced anti-tumor T cell responses (129). They promote tumor growth by iCP expression (CTLA-4, PD-1 and others) as well as production of IL-10 and TGF-β. An increased frequency of Tregs correlates with disease progression and metastasis in both experimental models and humans (130). CTLA-4 blockade expands the Treg frequency and high levels of soluble CD25 interleukin-2 receptor chair-alpha (IL2Rα) has been correlated with resistance to anti-CTLA-4 therapy (131). This was confirmed by Treg depletion potentiating the iCPI therapy (132). It has been suggested that early recruitment of Tregs to the TME inhibits an effective tumor response and lack of response to iCPI. PD-1 blockade with Nivolumab attenuated the activated T cell phenotypes during the course of therapy, promoted CTL proliferation and resistance to Treg-mediated suppression by down-regulating the intracellular expression of FoxP3, while Tregs increased during disease progression (133). An increased ratio of CTL to Treg in tumor tissues has been associated with response to CTLA-4 and PD-1 blockade.

### Natural Killer Cells as Players for Innate Immune Responses

Natural killer (NK) cells are effector cells of the innate immune system and important players in mounting innate anti-tumoral immune responses by their ability to directly target and eliminate viral infections as well as neoplastic transformed cells (134). Under pathological conditions and during inflammation, the NK cell activation depend on the balance between inhibitory as well as activating signals, which determine the NK cell mediated cytotoxicity. In addition, NK cells are involved in other immune regulatory processes and could modulate adaptive immune responses, since they share characteristics with adaptive lymphocytes (134). They could also interact with mast cells and effect tumorgenesis due to the production of pro-angiogenic factors and thus play an important role alone or in combination with mast cells in the regulation of angiogenesis (135). There is increasing evidence that NK cells are involved in regulating metastatic dissemination. NK cells are often shown to reduce metastatic efficacy of tumor cell lines *in vivo*, while low NK cell activity is correlated with advanced disease and metastasis formation (136, 137). Furthermore, the presence of tumor-infiltrating NK cells is a positive prognostic marker for multiple tumors (138–140).

### Tumor-Infiltrating Regulatory Myeloid Cells

Tumor-infiltrating myeloid cells comprise MDSCs, tumor-associated granulocytes, TAMs and DCs, generate and promote both immunogenic and tolerogenic responses (141–143). MDSCs are heterogeneous immune-suppressive immature myeloid cells that can be divided into a polymorphonuclear subset and a monocytic subset. They support tumor growth, epithelial to mesemchymal transition (EMT) and predict poor prognosis of patients, but their role in tumorgenesis has still to be defined (144, 145). MDSCs exert their effects by producing immune suppressive factors, like arginine 1 (Arg-1) expression, nitric oxide (NO), cyclooxygenase-2 (COX-2), reactive oxygen species (ROS), and activate Treg via CD40–CD40L interactions (146–148). In melanoma, elevated levels of CXCL17 were found, which recruits MDSCs and predicts non-responders to iCPI (149).

Tumor-associated neutrophils (TANs) and TAMs have been classified as an anti-tumor (type 1) or pro-tumor (type 2) phenotype. Pro-tumor effects of TANs include dampening of CTL response, increased angiogenesis, and modulation of cellular trafficking. Type 1 TAMs (M1) produce immune stimulatory cytokines, like IL-6, IL-12 and CXCL9, that promote recruitment of CTLs, while type 2 TAMs (M2) exhibit an immune suppressive signature and support tumor growth by release of angiogenic factors, like IL-10 and CCL22, matrix remodeling mediated by proteases, and by inhibition of CTL and DC activity (150–153). In addition, TAMs promote Tregs by inducing the skewing of blood-derived CD4^+^ T cells toward an immunosuppressive phenotype due to their decreased production of effector cytokines, increased IL-10 production and enhanced expression of the co-inhibitory molecules PD-1 and TIM-3 (154, 155). However, the interaction between TA-specific CD4^+^ Th1 cells and TAMs might shift the intra-tumoral M1/M2 ratio toward an M1 phenotype (155). PD-L1 expression of monocytes and TAMs promote immune evasion and correlate with disease progression in hepatocellular carcinoma. This might be mediated by a hypoxia inducible factor 1α induced increased expression of the receptor TREM1 in TMAs resulting in immune suppression mediated by Treg recruitment, which was associated with disease progression as well as resistance to anti-PD-L1 treatment (156). Fc-gamma receptors (FcγRs) expressed by M2 TAMs facilitate anti-tumor response to CTLA-4 inhibition through Treg depletion (157, 158). Tumor-infiltrating eosinophils promote infiltration of CTLs by polarization of TAMs and normalization of the tumor vasculature, and predict a better prognosis in colon cancer (159).

The heterogenic family of DCs, including classical (cDCs) and plasmacytoid DCs (pDCs), are antigen-presenting cells (APC) that prime and regulate CTL responses. Anti-viral immune responses rely heavily on pDC-derived type I IFNs, while pDCs in tumors exert immunosuppressive activities. In contrast, tumor-infiltrating cDC increase T cell activation in lung cancer and melanoma patients forming tertiary lymphoid clusters, which are associated with better outcomes (160, 161). Tertiary lymphoid clusters also correlated with improved survival in pancreatic cancer (162). The rare subgroup of CD103(+) (integrin αE)^+^ DCs are strong stimulators of CTL and dependent on different transcription factors, like IRF8, Zbtb46, and Batf3. These CD103 cDCs (Batf3-cDC, cDC1) are also associated with CTL and increased OS for patients with breast, head and neck or lung cancer (163). In lung adenocarcinoma murine models, immunogenic chemotherapy (oxaliplatin-cyclophosphamide) has been reported to up-regulate toll-like receptor 4 (TLR-4) on tumor-infiltrating Batf3-cDCs, which leads to the recruitment of CTLs and sensitization to iCPIs (164).

### Gut Microbiome, Immune Cell Interaction—iCPI Therapy

T cells as members of the adaptive immune system are involved in gut homeostasis, inflammation, and carcinogenesis (165). Recently, association between microbiota profiles, cancer susceptibility, and responsiveness to cancer therapy has been suggested (166–168). Indeed, microbiota could modify the immune response and influence the response to chemotherapy and immunotherapy (169, 170). Emerging evidence has suggested that the cross-talk between the gut microbiome and immune cells plays a role in determining responses to iCPI therapy. Indeed, the composition of the gut microbiome has been associated with response to iCPI in pre-clinical models as well as in patients. For example, in murine melanoma, commensal Bifidobacterium has been reported to promote the efficacy of anti-PD-L1 therapy by augmenting the function of DCs leading to CTL priming and infiltration (171). Recent studies in melanoma, lung, and kidney cancer patients have demonstrated an association of commensal gut microbiome with response to iCPI (172). Baseline gut microbiota enriched with Faecalibacterium and other Firmicutes is associated with a better response (173). In melanoma patients responding to iCPI more abundant species included Bifidobacterium, Collinsella, Enterococcus, Clostridiales, Rominococcus and Faecalibacterium, while low levels of Akkermansia muciniphila were observed in epithelial cancers not responding to iCPI (174). Patients with a favorable gut microbiome had increased expression of cytolytic T cell markers and APM components, and an increased ratio of CD8^+^ CTLs to FoxP3^+^CD4^+^ Tregs. Furthermore, metagenomic studies revealed functional differences in gut bacteria in responders. These are characterized by an enrichment of anabolic pathways and an enhanced systemic and antitumor immunity in responding patients with a favorable gut microbiome as well as in germ-free mice receiving fecal transplants from responding patients (172). Thus, the modulation of the components in the gut microbiome can augment anti-tumor immunotherapy. However, there exist several challenges including optimal composition of the gut microbiome and the therapeutic strategy to achieve that composition.

### Resistance to Checkpoint Inhibitors

Abnormalities of the HLA class I antigen and IFN signaling pathways often correlate with the development of resistances to various kinds of immunotherapies including iCPI treatment and adoptive cell therapy (ACT) (64, 175–178). These could be categorized into intrinsic and acquired immune resistance (177, 179, 180) and are associated with an altered tumor T cell interaction. An increased knowledge of these processes might lead to the reprogramming of the immunologically “cold” TME characterized by a low immune cell infiltration and low TCR diversity (181, 182) and an increased T cell function (183–185). Combining the modulation of the immune cell repertoire and the reduction of immune suppressive metabolites and cytokines of the TME and enhancement of T cell tumor interaction with iCPI and/or vaccinations or even targeted therapies are currently tested in diverse clinical trials (186, 187).

## Conclusions

There is strong evidence of an emerging role of T cell tumor interactions for the outcome of patients in general and regarding the efficacy of immunotherapies including iCPIs. The pathways involved in the regulation of the interaction between tumor and T cells are broad and highly dynamic. Tumors developed a plethora of adaptions leading to escape from counter-regulations of the immune system. This is mediated by ineffective T cell responses due to low tumor immunogenicity and the suppressive influence of the TME. The use of iCPI showed that the manipulation of inhibitory signaling pathways creates anti-tumoral immune responses. However, the efficacy of iCPIs is still limited. Thus, a better understanding of these processes might lead to the development of innovative therapies in order to reactivate T cell responses.

## Author Contributions

BS designed the project and wrote the manuscript.

### Conflict of Interest Statement

The author declares that the research was conducted in the absence of any commercial or financial relationships that could be construed as a potential conflict of interest.

## Abbreviations

AML: acute myeloid leukemia
APC: antigen-presenting cells
Arg-1: arginine 1
cDC: classical dendritic cell
COX-2: cyclooxygenase−2
CRC: colorectal carcinoma
CTL: cytotoxic lymphocytes
CTLA-4: T lymphocyte antigen 4
DC: dendritic cell
ECM: extracellular matrix
EMT: epithelial to mesenchymal transition
FcγR: Fc-gamma receptor
FDA: Food and Drug Administration
FOXP3: forkhead box P3
gzmb: granzyme B
HLA: human leukocyte antigen
HNSCC: head and neck squamous cell cancer
ICOS: inducible T cell costimulatory
iCPI: immune check point inhibitors
iCP: immune checkpoint
IFN: interferon
IL: interleukin
IL-2Rα: interleukin-2 receptor chain-alpha
LAG-3: lymphocyte activation gene-3
M1: type 1 TAM
M2: type 2 TAM
mAb: monoclonal antibody
MDSC: myeloid-derived-suppressor cell
MHC: major histocompatibility complex
MSI: microsatellite-instable
MSS: microsatellite-stable
NK: natural killer
NO: nitric oxide
NSCLC: non-small cell lung cancer
OS: overall survival
PD-1: programmed cell death protein 1
pDC: plasmacytoid dendritic cell
PD-L1: programmed cell death 1 ligand 1
RCC: renal cell cancer
ROS: reactive oxygen species
RT: radiation therapy
SCLC: small cell lung carcinoma
TAA: tumor associated antigen
TAF: tumor associated fibroblasts
TAM: tumor-associated macrophage
TAN: tumor-associated neutrophil
TCR: T cell receptor
Teff: effector T cell
Tex: exhausted CD8^+^ T cell
TGF-β: transforming growth factor-beta
TIGIT: T cell immunoglobulin and immunoreceptor tyrosine-based inhibitory motif
TIL: tumor infiltrating lymphocyte
TIM-3: T cell immunoglobulin and mucin domain containing protein-3
TLR-4: toll-like receptor 4
TMB: tumor mutational burden
TME: tumor microenvironment
Treg: regulatory T cell.
